# Health Status and Individual Care Needs of Disabled Elderly at Home in Different Types of Care

**DOI:** 10.3390/ijerph191811371

**Published:** 2022-09-09

**Authors:** Qi Tang, Min Yuan, Wenhui Wu, Huanyun Wu, Cao Wang, Gang Chen, Chengyue Li, Jun Lu

**Affiliations:** 1 School of Public Health, Fudan University, Shanghai 200032, China; 2 China Research Center on Disability, Fudan University, Shanghai 200032, China; 3 Key Laboratory of Health Technology Assessment, National Health Commission, Fudan University, Shanghai 200032, China; 4 Shanghai Jinshan District Health Service Management Center, Shanghai Jinshan District Municipal Health Commission, Shanghai 200540, China

**Keywords:** disability, elderly population, health status, care needs, types of care, quality of life

## Abstract

For the disabled, paying attention to their health status is the starting point to discovering their survival problems, while meeting their care needs is the end point to solving their survival problems. As the country with the largest number of disabled elderly in the world, how to ensure this group could obtain appropriate home care is a major public health issue facing China. Thus, we conducted a cross-sectional study from October to December 2020 to explore the basic characteristics and health status of disabled elderly in different types of care who are living at home in 37 streets in Shanghai, as well as the individual care needs and its relevance. We observed the significant differences in the number of diagnoses (*p* = 0.03), smoking (*p* = 0.009), drinking (*p* = 0.016), exercise (*p* = 0.001), activity of daily living (*p* < 0.0001), and the quality of life (*p* < 0.0001) across care types. The care needs of the disabled elderly are diversified, of which a vast majority of them have not been fully guaranteed. The urgent need for improving the identification accuracy of care needs of disabled elderly, as well as the development of elaborate and personalized care programs for them, is needed.

## 1. Introduction

According to the data of China Disabled Persons’ Federation (CDPF), there are about 85-million disabled people in China, and over 44-million disabled or partially disabled elderly people [1,2,3]. Compared with the general population, the disabled elderly have low health levels and high health risks, and are the most prominent group with health problems and in urgent need of care. Ensuring and promoting the survival and development of people with disabilities is not only the improvement and compensation of life functions at the individual level, but also the liberation of labor functions at the family and social level [4]. The Chinese government has issued a number of policies which call to focus on solving the health problems of key groups such as the disabled and the elderly, and to develop and ensure health services for them [5,6,7].

As one of the basic responsibilities of public health, population health status assessment is the basic guide for formulating health strategy and health planning [8]. Health-status assessment is the first step to determine the level and change of health, and it is a necessary way to obtain the current status of people with disabilities [9]. Care is a highly necessary special service for the disabled, which is mostly provided by the family and the government. Due to the existence of dysfunction, the disabled people inevitably have a certain degree of care needs, that is, the need for compensation behavior for missing or impaired functions [10]. Driven by this need, compensatory care services have become an important link to maintain the daily life of people with disabilities, which has an important life-support significance for them. Therefore, for the disabled people, paying attention to their health status is the starting point to discover their survival problems, while meeting their care needs is the end point to solve their survival problems. Visibly, the comprehensive assessment of their health status and care needs has become an important link in analyzing their health problems.

According to different places, the ways of accessing care services for the disabled elderly can be divided into home care, community care, and institutional care [11]. Because of the familiar family environment and lower economic cost, home care is the most important and common way to care for the disabled elderly, which plays a fundamental role in the long-term care system for the disabled elderly [12]. Thereinto, informal care refers to the care provided by spouses, children, and relatives on the basis of family affection for free, while formal care refers to the paid care services provided by professionals, teams, or institutions [13]. As the country with the largest absolute number of disabled elderly in the world, how to ensure that the disabled elderly could obtain appropriate home care is a major public health and social problem facing China.

Shanghai is a typical megacity in China with a developed economy, of which the population-aging level and the number of disabled elderly ranked top in China [14]. According to the latest official data, there were 385,747 disabled elderly aged 60 and above in Shanghai in 2019, growing at a compound annual growth rate (CAGR) of 16.94% between 2015 and 2019 [15]. Shanghai is the first city in China to build a care system for the disabled elderly, with relatively sufficient care resources [16]. The home care status of the disabled elderly in Shanghai basically represents the highest level that can be reached in China at present, and the care needs of the elderly are also the most diverse. Therefore, an assessment of the health status and care needs of the disabled elderly at home in Shanghai could find the key targets for further improving the home care service system in Shanghai, and also provide experience and theoretical support for developing regions. Given these situations, this study explored the basic characteristics and health status of disabled elderly in different types of care, as well as the individual care needs and its relevance. In order to understand the care status and the satisfaction of care needs of the disabled elderly, providing the data and reference for policy formulation.

## 2. Materials and Methods

### 2.1. Study Design

A cross-sectional study on health status and individual care needs of disabled elderly at home was conducted from October to December 2020 in Shanghai. Considering the number of disabled elderly people and the degree of cooperation with the team, the three districts of Yangpu District, Hongkou District, and Jinshan District in Shanghai were selected as the research areas. Taking the streets under the jurisdiction of the three districts as the basic unit. The cluster sampling method was adopted, and finally the disabled elderly living at home in 37 streets were selected as the survey subjects. The respondents were summoned to the office of the Disabled Persons’ Federation of the street, and the investigators conducted one-to-one and face-to-face interviews with the respondents. For the respondents who were unable to go out to the gathering point due to physical difficulties, two investigators were selected as a group to conduct the household survey. The questionnaire [17] consisted of four parts: basic characteristics (gender, age, household registration, marital status, education, occupational status, annual household income, source of income, basic medical insurance type, BMI, disability type [18], disability severity, disability duration, and type of care), health status (disease status and health behaviors), health outcomes (daily living activity, mental health, and quality of life), and individual care needs.

This study was approved by the Ethics Committee of School of Public Health, Fudan University (International Registration Number: IRB00002408 & FWA00002399; approval number: IRB#2015-08-0563).

### 2.2. Patients

The disabled elderly aged 60 years and above with household registration in 37 streets in Shanghai were selected as subjects in this study.

The inclusion criteria are as follows: (i) aged 60 and above; (ii) holding the People’s Republic of China Disabled Person’s Certificate; (iii) the disabled elderly or their main caregivers (family members) can answer the questionnaire. Those who can answer by themselves shall complete the questionnaire by themselves. For the disabled elderly who can not answer by themselves, their family members shall complete the questionnaire; and (iv) voluntarily participate in projects and obtain informed consent.

People with the following conditions were excluded: (i) age less than 60; (ii) none of the People’s Republic of China Disabled Person’s Certificate; (iii) communication difficulties with disabled elderly and their family members.

In this study, 600 questionnaires were distributed and 586 were recovered (the recovery rate was 97.67%), among which 559 were effective (the effective rate was 93.17%).

### 2.3. Measures and Variables

#### 2.3.1. Health Status

Including the measures of disease status (chronic disease, the number of diagnoses, and main disease) and health behaviors (smoking, drinking, and taking exercise).

#### 2.3.2. Health Outcomes

(i) Activity of daily living (ADL): The combination scale of Activity of Daily Living Scale and Instrumental Activity of Daily Living (ADL-IADL) was used to evaluate the ADL of the disabled elderly (a total of 14 items) [19,20,21,22]. A total of 0~16 points means having self-care ability; 17~21 means function decline; and 22~56 means dysfunction.

(ii) Mental health: The General Health Questionnaire (GHQ)−12 was used to evaluate the mental health of the disabled elderly (a total of 14 items) [23,24,25]. A total of 0~15 points means low-risk; 16~20 means medium-risk; and 21~36 means high-risk.

(iii) Quality of life (QOL): The SF−12 was used to evaluate the QOL of the disabled elderly, including two sub-scales of Physical Component Summary (PCS) and Mental Component Summary (MCS) [26,27].

#### 2.3.3. Individual Care Needs

The care needs from the disabled elderly included 14 care services items, including basic living support (BLS), Medical and nursing support (MNS), social participation support (SPS), and psychological support (PS). Relationship networks between the care needs of the disabled elderly were investigated by Social Network Analysis (SNA).

### 2.4. Statistical Analysis

IBM SPSS Statistics (version 23.0, Armonk, New York, NY, USA) was used for data analysis. The data normality test was performed on the measurement data; the mean ± standard deviation was used to describe the measurement data with normal distribution, and the composition ratio (%) was used to describe the count data. The differences between groups were analyzed by Analysis of Variance (ANOVA) for normally distributed measures, non-parametric tests for non-normally distributed measures, and chi-square test and Fisher exact probability method for counting data. Logistic regression analysis was used to analyze the influence factors. A *p* value < 0.05 was considered significant. The UCINET (version 6, Stephen Borgatti, Irvine, CA, USA) and NetDraw (UCINET’s bundled software, Harvard, MA, USA) were used for the SNA.

## 3. Results

### 3.1. Basic Characteristics of the Disabled Elderly

Five-hundred-and-fifty-nine disabled elderly were included in our study. Among these disabled elderly at home, 313 (56.0%) were male and 246 (44.0%) were female. There were 337 (60.29%) disabled elderly aged between 60 and 69, and 101 (18.06%) aged over 80. BMI was mainly 18.5 to 23.9 kg/m^2^ (50.1%). In terms of marital status, 385 disabled elderly (68.9%) were married. It is worth noting that 84 (15.0%) of the disabled elderly are unmarried, which means that they have difficulty in obtaining support from informal care. In terms of monthly household income, 329 (58.9%) had a monthly income of USD 450 to USD 749, and 178 (31.8%) disabled elderly had a monthly income of less than USD 450 including 15 (2.7%) poor disabled elderly with less than USD 150. These elderly are likely to have difficulty purchasing formal care services on their own. A total of 193 (34.5%) disabled elderly at home used self-care, 139 (24.9%) relied on family care, and most of the elderly (40.6%) obtained care services through the combination of informal care and formal care. Table 1 summarizes the basic characteristics of the disabled elderly in our study.

### 3.2. Disease Status and Health Behaviors of Disabled Elderly in Different Types of Care

Table 2 summarizes the disease status and health behaviors of disabled elderly in different types of care. The disabled elderly who rely on family care and public care suffer more from chronic diseases (52%), but there is no statistical difference among different groups. In the number of diagnoses, smoking, drinking, and taking exercise, there were statistical differences among the disabled elderly in different types of care.

### 3.3. Health Outcomes of Disabled Elderly in Different Types of Care

Table 3 presented the health outcomes of disabled elderly in different types of care.

According to the measured results of the ADL-IADL scale, 46 (8.2%) disabled elderly were in a self-care state, 145 (26.0%) were in a functionally declining state, and 368 (65.8%) were in a dysfunctional state. There were statistically significant differences in the ADL scores of disabled elderly with different types of care (*p* < 0.0001).

According to the measurement results of GHQ-12 scale, 479 (85.7%) disabled elderly at a low-risk level of mental health, 38 (6.8%) at a medium-risk level, and 42 (7.5%) at a high-risk level. There was no statistical difference among the groups.

The QOL of the disabled elderly at home was measured by the SF-12 scale. Higher scores indicate better health QOL. The results showed that the PCS and MCS scores of self-care disabled elderly at home were higher than those of the other two groups, and the differences among the groups were statistically significant (*p* < 0.0001).

### 3.4. Individual Care Needs for Disabled Elderly in Different Types of Care

Among the 559 disabled elderly included in this study, the highest rate of care needs was economic supplement (BLS−3) (78.2%), and the second was housekeeping (BLS−1) (77.8%). In general, the care needs of disabled elderly at home were concentrated in BLS and MNS, while the needs for SPS and PS were relatively low (Table 4).

The rate of satisfaction of individual care needs for the disabled elderly at home is generally low, and no care service exceeds 30% (Table 5). In general, regardless of the types of care obtained, the care needs of the vast majority of disabled elderly at home are still not fully met.

**Figure 1:**
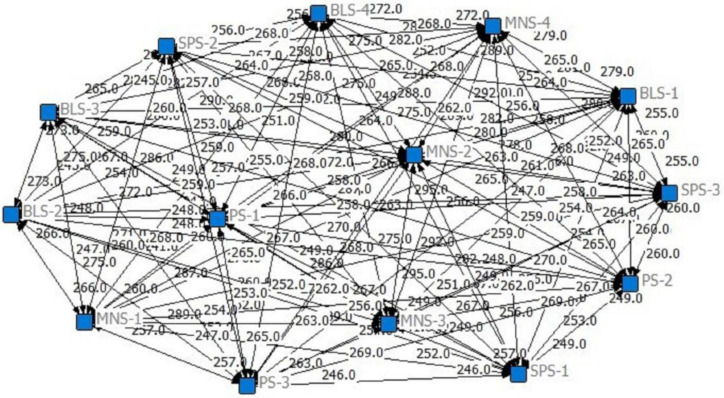
Relationship networks between the inadequately met care needs of the disabled elderly. Netdraw software was used for visual processing to form the relationship network between the inadequately met care needs of the disabled elderly. It can be seen that the care service items that are not fully satisfied in the network are basically the same in centrality. The items with high correlation mainly included Housekeeping (BLS−1), Economic component (BLS−3), Housing Retrofit (BLS−4), Escort to Hospital (MNS−1), Medical Service (MNS−2), Care and Rehabilitation (MNS−3), and Emergency rescue (MNS−4).

Table 6 shows the correlation matrix between the disabled elderly at home who are not fully satisfied with their care needs displayed by UCINET data processing software. Netdraw software was used for visual processing to form the relationship network between the inadequately met care needs of the disabled elderly (Figure 1). It can be seen that the care service items that are not fully satisfied in the network are basically the same in the Centrality. The items with high correlation mainly included Housekeeping (BLS−1), Economic component (BLS−3), Housing Retrofit (BLS−4), Escort to Hospital (MNS−1), Medical Service (MNS−2), Care and Rehabilitation (MNS−3), and Emergency rescue (MNS−4). This indicates that the unmet care needs of BLS and MNS for the disabled elderly at home are generally highly correlated in the network, which means that giving priority to fully meeting these two types of care needs would have the best driving effect on meeting the care needs of other home-disabled elderly.

## 4. Discussion

Care is one of the core needs of people in their later years [28]. Obtaining appropriate care services is the key to maintaining the health status of the elderly and improving their QOL [29,30]. Comparing the elderly with relatively healthy and stable physical functions, the disabled elderly affected by congenital or acquired factors have obviously stronger need for care services, and higher requirements for the content and quality of care services [31,32]. This means that for a long time in the future, China may need to incline the limited care resources to the disabled elderly to ensure the basic QOL of the extremely vulnerable population [33]. Obviously, the QOL and care needs of the disabled elderly are still not fully guaranteed, and their care status is still not optimistic.

Shanghai has developed and implemented a number of care policies including long-term care insurance (LTCI) [34], and has been vigorously developing social care resources such as care services embedded in community [35,36] and multi-family cooperative care services [37]. Nevertheless, the results of this study show that the disabled elderly who use both formal care and informal care still account for the majority. On the one hand, under the influence of Chinese traditional culture, it is regarded as a family responsibility for spouses and children to take care of the disabled elderly. The vast majority of the disabled elderly are more eager for the company of their families and are used to being taken care of by their relatives. On the other hand, the disabled elderly have high requirements on the length and continuity of care-service supply, and some severely disabled elderly even need 24 h care. However, the formal care resources available cannot meet these needs. It can be seen that whether formal care and informal care can complement each other in terms of service time and service content is the key to the quality of care [38].

From the results of this study, it can be seen that the QOL of the disabled elderly is not high in both PSC and MCS scores [39], and the elderly in self-care have a higher score (*p* < 0.001). While this result may be due to the poor health basis of the elderly requiring formal care, it also suggests that the care currently available to these disabled elderly is still very limited in its effect on improving their QOL. Theoretically, formal care can meet the needs of the disabled elderly in living care, while informal care can provide them with psychological support [40,41]. However, from the results of this study, it is possible that due to the lack of synergy between formal care and informal care, the effect of care services on the QOL of the disabled elderly may still be limited. It is particularly important to strengthen the living care services and pay attention to the psychological support needs of the disabled elderly, so as to improve the quality of care for the disabled elderly and enhance their family development potential [42,43].

The study shows that the care needs of the vast majority of disabled elderly have not been fully met, indicating that there is still a large gap of care resources for the disabled elderly under the current policy environment. The first, at present, the care resources that can provide for the disabled elderly are still insufficient in terms of the comprehensiveness of the service content, which can only provide very limited service items and insufficient daily service time. Due to the lack of human resources and the low ability of formal caregivers, it is difficult to provide the services required by the disabled elderly, such as accompanying medical treatment, auxiliary equipment rental, psychological support, and home diagnosis and treatment. As a result, there is a high degree of overlap with service homogeneity between formal care and informal care for the disabled elderly [44]. The disabled elderly do not have a strong perception of formal care, and their caregivers do not recognize the support of formal care, leading to a large number of formal care resources not fully playing a role [45]. The second, some care resources have great defects in the allocation process. When assessing the care needs of the disabled elderly, it is often based on the results of one observation, rather than the multi-dimensional surveys on the health status, disability, and care needs by family doctors, health records and community workers, leading to the risk of large inaccuracy in the assessment results. The contents of care services provided by some disabled elderly people are highly overlapped, and the care needs of the disabled elderly who most need care resources are difficult to be met.

This study suggests that the current situation of care for the disabled elderly is still not optimistic, and there is still great room for development and improvement of the care policy. As a pioneer city in the development of care for the elderly in China, Shanghai should be recognized for its efforts in the development of care resources, but it should not ignore the various problems faced in care for the disabled elderly. In the future development of elderly care in Shanghai and even China, we should further perfect the evaluation system and mechanism of care needs, improving the accuracy of identification of care needs of disabled elderly and their informal caregivers [46]. At the same time, it is necessary to clearly distinguish the difference between the content of care services needed by the disabled elderly and the ordinary elderly. Medical institutions can mark the key points of care needed between the disabled elderly and the ordinary elderly in the process of diagnosis and treatment, so as to break the information barrier and promote the transmission of information of the disabled elderly, such as the disease situation and care points, to the community healthcare centers. At the community level, more elaborate and personalized care programs should be developed for the disabled elderly [47,48,49,50]. From the three levels of individual, family, and community, the disabled elderly should be guaranteed continuous access to appropriate care [51,52].

The current study had several limitations. Firstly, information on exposure and outcomes was obtained through a self-reported questionnaire, which may constitute a reporting bias. However, this is likely to be an undifferentiated bias. Secondly, this study lacks situation analysis of caregivers for the disabled elderly, who play an important role in influencing the health status and care needs of them. We will pay more attention to this key group in the subsequent research.

## 5. Conclusions

In conclusion, this study showed differences in the health status and individual care needs of disabled elderly at home based on types of care. Our findings revealed that the QOL of the disabled elderly is low, of which those in self-care are higher than those with formal or informal care, and the care needs of the vast majority of the disabled elderly have not been fully guaranteed. The urgent need for improving the identification accuracy of care needs of disabled elderly, as well as development of elaborate and personalized care programs for them, is called for.

## Figures and Tables

**Table 1:** Basic characteristics of the disabled elderly.

Characteristics	Participants (*n* = 559)		Characteristics	Participants (*n* = 559)	
	*n*/mean	%/SD		*n*/mean	%/SD
Gender			Source of income		
Male	313	56.0	Pension or Annuity	485	86.8
Female	246	44.0	Government or social relief	50	8.9
Age (year) ^1^	70.2	8.3	Family support	24	4.3
Age group			Basic medical insurance type		
60~69	337	60.3	for urban workers	305	54.6
70~79	121	21.6	for residents	254	45.4
≥80	101	18.1	BMI (Kg/m^2^) ^1^	23.4	3.8
Household registration			BMI group		
Rural	95	17.0	<18.5	48	8.6
Urban	464	83.0	18.5~23.9	280	50.1
Marital Status			24~27.5	171	30.6
Married	385	68.9	≥27.5	60	10.7
Unmarried	84	15.0	Disability type		
Divorced or widowed	90	16.1	Visual	64	11.4
Education			Hearing and Speech	51	9.1
Primary School or below	174	31.1	Physical	327	58.5
Middle School	220	39.4	Intellectual	76	13.6
High School or Technical Secondary School	117	20.9	Mental	30	5.4
			Multiple	11	2.0
Junior College or above	48	8.6	Disability severity		
Occupational Status			Level 1	130	23.3
On the job	55	9.8	Level 2	241	43.1
Retirement	463	82.8	Level 3	105	18.8
Unemployed	41	7.4	Level 4	83	14.8
Monthly household income ($)			Disability duration (year) ^1^	29.4	23.5
<150	15	2.7	Type of care		
150~449	163	29.1	Self-care	193	34.5
450~749	329	58.9	Family-care	139	24.9
≥750	52	9.3	Family-care & Public-care	227	40.6

^1^ The data in this marked part are presented as mean (SD), and the others as frequencies (%).

**Table 2:** Disease status and health behaviors of disabled elderly in different types of care.

Variables	Total (*n* = 559)	Type of Care			*p* Value
		Self-Care (*n* = 193)	Family-Care (*n* = 139)	Family-Care & Public-Care (*n* = 227)	
Chronic disease					0.10
yes	261 (46.7)	81 (42.0)	62 (44.6)	118 (52.0)	
no	298 (53.3)	112 (58.0)	77 (55.4)	109 (48.0)	
Number of diagnoses ^1^	1.0 (1.3)	0.9 (1.2)	1.0 (1.4)	1.2 (1.5)	0.03
Main disease					
hypertension	178 (31.8)	56 (29.0)	44 (31.7)	78 (34.4)	0.50
diabetes	81 (14.4)	23 (11.9)	16 (11.5)	42 (18.5)	0.08
COPD ^2^	5 (0.8)	2 (1.0)	1 (0.7)	2 (0.9)	0.95
chronic pneumonia	4 (0.7)	1 (0.5)	1 (0.7)	2 (0.9)	0.91
chronic bronchitis	31 (5.5)	9 (4.7)	13 (9.4)	9 (4.0)	0.07
CHD ^3^	40 (7.1)	11 (5.7)	11 (7.9)	18 (7.9)	0.63
cerebral hemorrhage	6 (1.0)	0 (0)	1 (0.7)	5 (2.2)	0.08
cerebral infarction	43 (7.6)	10 (5.2)	7 (5.0)	26 (11.5)	0.02
cataract	52 (9.3)	12 (6.2)	19 (13.7)	21 (9.3)	0.07
glaucoma	7 (1.2)	2 (1.0)	3 (2.2)	2 (0.9)	0.54
arthritis	41 (7.3)	12 (6.2)	6 (4.3)	23 (10.1)	0.09
rheumatism	17 (3.0)	1 (0.5)	7 (5.0)	9 (4.0)	0.03
advanced tumor	10 (1.7)	2 (1.0)	1 (0.7)	7 (3.1)	0.16
Smoking					0.009
yes	87 (15.6)	42 (21.8)	20 (14.4)	25 (11.0)	
no	472 (84.4)	151 (78.2)	119 (85.6)	202 (89.0)	
Drinking					0.016
yes	68 (12.2)	34 (17.6)	14 (10.1)	20 (8.8)	
no	491 (87.8)	159 (82.4)	125 (89.9)	207 (91.2)	
Taking exercise					0.001
yes	182 (32.6)	82 (42.5)	39 (28.1)	61 (26.9)	
no	377 (67.4)	111 (57.5)	100 (71.9)	166 (73.1)	

^1^ The data in this marked part are presented as mean (SD), and the others as frequencies (%). ^2^ COPD: chronic obstructive pulmonary disease. ^3^ CHD: coronary heart disease.

**Table 3:** Health outcomes of disabled elderly in different types of care.

Variables	Total (*n* = 559)	Type of Care			*p* Value
		Self-Care (*n* = 193)	Family-Care (*n* = 139)	Family-Care & Public-Care (*n* = 227)	
ADL ^1^					
Total points ^2^	29.1 (11.5)	22.1 (6.5)	27.6 (9.1)	36.0 (12.3)	<0.0001
Ranking points					<0.0001
Self-care ability	46 (8.2)	32 (16.6)	6 (4.3)	8 (3.5)	
Function declines	145 (26.0)	78 (40.4)	37 (26.6)	30 (13.2)	
Dysfunction	368 (65.8)	83 (43.0)	96 (69.1)	189 (83.3)	
Mental health (GHQ−12 ^3^)					
Total points ^2^	7.5 (8.1)	8.1 (6.6)	8.0 (8.1)	6.7 (9.3)	0.006
Ranking points					0.05
Low-risk	479 (85.7)	175 (90.7)	115 (82.7)	189 (83.3)	
Medium-risk	38 (6.8)	11 (5.7)	13 (9.4)	14 (6.2)	
High-risk	42 (7.5)	7 (3.6)	11 (7.9)	24 (10.5)	
Quality of life (SF−12 ^4^)					
PCS ^2^	32.8 (8.8)	37.6 (8.5)	30.7 (7.7)	30.0 (8.0)	<0.0001
MCS ^2^	46.5 (11.8)	50.3 (10.3)	46.7 (12.0)	43.2 (12.1)	<0.0001

^1^ ADL: Activity of daily living. A total of 0~16 points means having self-care ability; 17~21 means function declines; and 22~56 means dysfunction. ^2^ The data in this marked part are presented as mean (SD), and the others as frequencies (%). ^3^ GHQ-12: General Health Questionnaire. A total of 0~15 points means low-risk; 16~20 means medium-risk; and 21~36 means high-risk. ^4^ SF−12 involved in calculating the SF−12 Physical Component Summary (PCS) and Mental Component Summary (MCS) scores.

**Table 4:** Individual care needs for disabled elderly in different types of care.

Care Services Items	Total (*n* = 559)	Type of Care			*p* Value
		Self-Care (*n* = 193)	Family-Care (*n* = 139)	Family-Care & Public-Care (*n* = 227)	
Basic living support					
Housekeeping (BLS−1)	435 (77.8)	133 (68.9)	112 (80.6)	190 (83.7)	0.001
Physical Care (BLS−2)	415 (74.2)	132 (68.4)	101 (72.7)	182 (80.2)	0.02
Economic subsidy (BLS−3)	437 (78.2)	134 (69.4)	112 (80.6)	191 (84.1)	0.001
Housing retrofit (BLS−4)	418 (74.8)	132 (68.4)	105 (75.5)	181 (79.7)	0.02
Medical and nursing support					
Escort to hospital (MNS−1)	419 (75.0)	134 (69.4)	105 (75.5)	180 (79.3)	0.06
Medical service (MNS−2)	426 (76.2)	134 (69.4)	108 (77.7)	184 (81.1)	0.01
Care and Rehabilitation (MNS−3)	420 (75.1)	133 (68.9)	104 (74.8)	183 (75.1)	0.02
Emergency rescue (MNS−4)	433 (77.5)	135 (69.9)	114 (82.0)	184 (81.1)	0.008
Social participation support					
Assistive devices rental (SPS−1)	388 (69.4)	132 (68.4)	99 (71.2)	157 (69.2)	0.85
Public activity space (SPS−2)	404 (72.3)	134 (69.4)	106 (76.3)	164 (72.2)	0.39
Recreational activities (SPS−3)	399 (71.4)	134 (69.4)	104 (74.8)	161 (70.9)	0.55
Psychological support					
Phone or home visit (PS−1)	403 (72.1)	134 (69.4)	99 (71.2)	170 (74.9)	0.45
Interactive activities (PS−2)	396 (70.8)	133 (68.9)	98 (70.5)	165 (72.7)	0.69
Psychological counseling by hotline (PS−3)	391 (69.9)	132 (68.4)	95 (68.3)	164 (72.2)	0.62

**Table 5:** Satisfaction of individual care needs for disabled elderly in different types of care.

Care Services Items	Total	Type of Care			*p* Value
		Self-Care	Family-Care	Family-Care & Public-Care	
Basic living support					
Housekeeping (BLS−1)	91 (20.9)	32 (24.1)	22 (19.6)	37 (19.5)	0.57
Physical Care (BLS−2)	91 (21.9)	32 (24.2)	17 (16.8)	42 (23.1)	0.35
Economic subsidy (BLS−3)	85 (19.5)	32 (23.9)	22 (19.6)	31 (16.2)	0.23
Housing retrofit (BLS−4)	76 (18.2)	32 (24.2)	19 (18.1)	25 (13.8)	0.06
Medical and nursing support					
Escort to hospital (MNS−1)	85 (20.3)	32 (23.9)	18 (17.1)	35 (19.4)	0.41
Medical service (MNS−2)	84 (19.7)	32 (23.9)	20 (18.5)	32 (17.4)	0.33
Care and Rehabilitation (MNS−3)	76 (18.1)	31 (23.3)	17 (16.3)	28 (15.3)	0.16
Emergency rescue (MNS−4)	80 (18.5)	31 (23.0)	19 (16.7)	30 (16.3)	0.27
Social participation support					
Assistive devices rental (SPS−1)	74 (19.1)	31 (23.5)	22 (22.2)	21 (13.4)	0.06
Public activity space (SPS−2)	74 (18.3)	32 (23.9)	20 (18.9)	22 (13.4)	0.07
Recreational activities (SPS−3)	74 (18.5)	32 (23.9)	20 (19.2)	22 (13.7)	0.08
Psychological support					
Phone or home visit (PS−1)	78 (19.4)	31 (23.1)	22 (22.2)	25 (14.7)	0.13
Interactive activities (PS−2)	71 (17.9)	31 (23.3)	18 (18.4)	22 (13.3)	0.08
Psychological counseling by hotline (PS−3)	70 (17.9)	31 (23.5)	18 (18.9)	21 (12.8)	0.06

**Table 6:** Incident matrix between the inadequately met care needs of the disabled elderly.

Care Services Program Code	BLS−1	BLS−2	BLS−3	BLS−4	MNS−1	MNS−2	MNS−3	MNS−4	SPS−1	SPS−2	SPS−3	PS−1	PS−2	PS−3
BLS−1	313	282	290	281	278	280	282	279	249	258	255	258	260	256
BLS−2	282	289	273	267	266	272	271	265	241	245	247	248	248	247
BLS−3	290	273	319	284	275	280	286	282	249	265	261	259	259	254
BLS−4	281	267	284	303	268	275	275	272	249	256	252	255	254	252
MNS−1	278	266	275	268	301	287	289	288	252	260	260	260	262	257
MNS−2	280	272	280	275	287	304	295	289	256	264	263	266	265	263
MNS−3	282	271	286	275	289	295	309	292	257	268	267	267	267	263
MNS−4	279	265	282	272	288	289	292	312	256	268	265	268	264	262
SPS−1	249	241	249	249	252	256	257	256	265	251	253	249	249	246
SPS−2	258	245	265	256	260	264	268	268	251	284	268	257	259	253
SPS−3	255	247	261	252	260	263	267	265	253	268	278	258	260	254
PS−1	258	248	259	255	260	266	267	268	249	257	258	282	270	265
PS−2	260	248	259	254	262	265	267	264	249	259	260	270	279	269
PS−3	256	247	254	252	257	263	263	262	246	253	254	265	269	273

## Data Availability

Not applicable.
